# Effect of a Health Belief Model Based Education Program on Women’s Cervical Cancer Knowledge and HPV Vaccination Attitudes in Primary Care Settings

**DOI:** 10.3390/healthcare14142118

**Published:** 2026-07-15

**Authors:** Mehmet Uçar, Muhammet Faruk Yiğit, Sibel Akgül Kartal, Adem Yağan

**Affiliations:** 1 Department of Medical Services and Techniques, Varto Gıyasettin Bingöl Vocational School, Muş Alparslan University, 49250 Muş, Türkiye; 2 Department of Nursing, Faculty of Health Sciences, Van Yuzuncu Yil University, 65040 Tuşba, Türkiye; muhammetfarukyigit@yyu.edu.tr; 3 Department of Midwifery, Faculty of Health Sciences, Van Yuzuncu Yil University, 65040 Tuşba, Türkiye; sibelakgulkartal@yyu.edu.tr; 4 Department of Family Medicine, Faculty of Medicine, Van Yuzuncu Yil University, 65040 Tuşba, Türkiye; admygn@icloud.com

**Keywords:** cervical cancer, HPV vaccine, women, health belief model, primary healthcare services, health education

## Abstract

**Highlights:**

**What are the main findings?**

- A Health Belief Model–based education program significantly increased women’s cervical cancer knowledge levels compared with the control group.
- The intervention significantly improved attitudes and beliefs toward HPV vaccination, while reducing perceived barriers and uncertainty regarding HPV vaccination.

**What are the implications of the main findings?**

- Structured educational interventions implemented in primary healthcare settings may strengthen women’s preventive health behaviors related to cervical cancer and HPV vaccination.
- Family Health Centers can play an important role in increasing HPV vaccine awareness and supporting cervical cancer prevention through theory-based education programs.

**Abstract:**

**Background/Objectives**: This study was conducted to determine the effect of a Health Belief Model–based education program implemented in Family Health Centers on women’s cervical cancer knowledge and their attitudes and beliefs toward HPV vaccination. **Methods**: This quasi-experimental pretest–posttest control group study was conducted between March and May 2026 at the Tuşba Training Family Health Center in Van, eastern Türkiye. The study included 150 women aged 18–49 years who had adolescent daughters aged 10–18 years. Data were collected using the Descriptive Information Form, the Cervical Cancer Knowledge Scale, and the Carolina HPV Immunization Attitudes and Beliefs Scale. The intervention group received a structured education program based on the Health Belief Model focusing on cervical cancer, HPV infection, and HPV vaccination. Descriptive statistics, chi-square tests, independent samples *t*-tests, paired samples *t*-tests, and mixed-design analysis of variance (ANOVA) were used for data analysis. **Results**: Following the educational intervention, cervical cancer knowledge scores increased from 2.13 ± 0.79 to 7.24 ± 0.84 in the intervention group, whereas no significant change was observed in the control group (*p* < 0.001). Total CHIAS scores increased from 24.32 ± 5.30 to 41.23 ± 2.51, accompanied by significant improvements in HPV vaccination attitudes and beliefs (*p* < 0.001). Significant Group × Time interaction effects were observed for cervical cancer knowledge, total CHIAS scores, and all CHIAS subdimensions (*p* < 0.001). **Conclusions**: The findings indicated that the Health Belief Model–based education program was associated with improved knowledge about cervical cancer and HPV and with more favorable attitudes and beliefs toward HPV vaccination. Structured educational programs implemented in primary healthcare settings may support the promotion of preventive health behaviors.

## 1. Introduction

Cervical cancer remains one of the most common cancers in women worldwide, although it is largely preventable through effective screening programs and vaccination. According to World Health Organization data, approximately 660,000 new cases of cervical cancer and 350,000 deaths were reported in 2022 [1]. The burden of the disease is particularly higher in low- and middle-income countries, which is associated with inequalities in access to screening and vaccination services [2]. High-risk Human Papilloma Virus (HPV) types are responsible for approximately 99% of cervical cancers, and HPV infection is considered the primary etiological factor of the disease [3].

The HPV vaccine is one of the most effective primary prevention methods for cervical cancer. Long-term follow-up studies show that the HPV vaccine significantly reduces the incidence of infection, precancerous lesions, and cervical cancer [4]. However, despite the safety and effectiveness of the HPV vaccine, vaccination rates worldwide are not at the desired level [5]. The main reasons for low vaccine acceptance rates include lack of information, misconceptions, concerns about vaccine safety, and sociocultural factors [6]. The insufficient level of knowledge among women about HPV and cervical cancer can negatively affect their attitudes and behaviors towards the vaccine [6,7].

Screening programs, which are among the secondary prevention methods in the prevention of cervical cancer, are of great importance. Early diagnosis is possible with Pap smear and HPV tests, and mortality rates can be significantly reduced [8]. However, it is reported that the participation rates of women in screening programs are insufficient in Türkiye, as in many other countries [8,9]. The main reasons for this situation include lack of information, low health literacy, and negative beliefs about preventive health behaviors [10].

One of the most commonly used theoretical models in explaining health behaviors is the Health Belief Model (HBM), which explains individuals’ health-related behaviors through the dimensions of perceived sensitivity, perceived seriousness, perceived benefit, and perceived barriers [11]. This model suggests that individuals develop behaviors based on their perception of the risk of contracting a disease and a cost–benefit assessment of protective behaviors. In the literature, it has been shown that the components of the HBM are important determinants of cervical cancer screening behaviors and HPV vaccine acceptance [12].

Recent studies have shown that HBM-based education programs are effective in increasing women’s knowledge levels and positively changing their health behaviors [13]. Primary healthcare services, in particular, are an important level of health where continuous contact with individuals is ensured, trust relationships are strong, and educational interventions can be effectively implemented.

In Türkiye, HPV vaccination is not yet included in the national immunization program, and awareness regarding HPV infection, cervical cancer prevention, and HPV vaccination remains limited among many women. Although cervical cancer screening services are offered free of charge through Family Health Centers, participation rates remain below desired levels. Family Health Centers serve as the first point of contact with the healthcare system for many women and therefore provide a valuable setting for preventive health education. In this context, theory-based educational interventions delivered in primary care settings may play an important role in improving cervical cancer awareness and promoting positive attitudes toward HPV vaccination among Turkish women.

The World Health Organization’s cervical cancer elimination strategy emphasizes HPV vaccination, cervical cancer screening, and timely treatment as key components of prevention. The WHO recommends that 90% of girls be fully vaccinated against HPV by age 15, 70% of women be screened with a high-performance test by ages 35 and 45, and 90% of women with cervical disease receive appropriate treatment. The WHO also recommends starting regular cervical cancer screening at age 30 years among the general population of women [1,2,7].

However, studies that evaluate the combined effect of structured HBM-based education interventions implemented in Family Health Centers on both women’s cervical cancer knowledge levels and their attitudes and beliefs towards the HPV vaccine are limited.

Despite the availability of effective cervical cancer screening and HPV vaccination strategies, insufficient knowledge, persistent vaccine-related misconceptions, and limited theory-based educational interventions in primary healthcare settings continue to hinder preventive health efforts among women.

In this context, the aim of this research is to determine the effect of an HBM-based education program implemented in Family Health Centers on women’s cervical cancer knowledge level and their attitudes and beliefs towards the HPV vaccine.

### Research Hypotheses

**H1.**  *An HBM-based education program is associated with increased women’s knowledge about cervical cancer.*

**H2.**  *An HBM-based education program is associated with more favorable attitudes and beliefs toward HPV vaccination.*

## 2. Materials and Methods

### 2.1. Research Design

**Figure 1:**
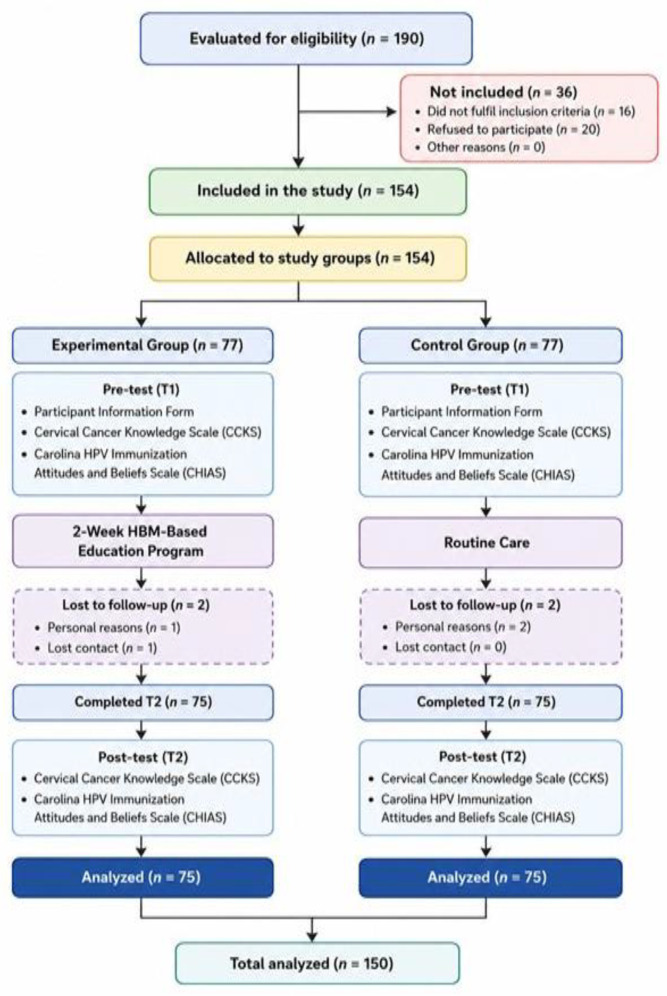
TREND Flow Diagram of Participant Recruitment, Allocation, Follow-Up, and Analysis. CCKS = Cervical Cancer Knowledge Scale; CHIAS = Carolina HPV Immunization Attitudes and Beliefs Scale.

This research was conducted using a quasi-experimental pretest–posttest control group design to evaluate the effect of an HBM-based education program implemented in Family Health Centers on women’s cervical cancer knowledge level and their attitudes and beliefs towards the HPV vaccine. The study was reported in accordance with the TREND (Transparent Reporting of Evaluations with Nonrandomized Designs) guideline for non-randomized intervention studies (Figure 1). The study was registered at ClinicalTrials.gov (Identifier: NCT07521163).

### 2.2. Location and Timing of the Study

The study was conducted at the Tuşba Training Family Health Center located in the Tuşba district of Van Province in eastern Türkiye. The data collection and implementation processes of the study were carried out between March 2026 and May 2026.

### 2.3. Population and Sample

The population of the study consisted of women aged 18–49 who applied to the Van Tuşba Training Family Health Center during the dates the study was conducted. A total of 154 women were enrolled in the study and allocated to the intervention group (n = 77) or the control group (n = 77). During follow-up, four participants were lost (three for personal reasons and one due to loss of contact). Therefore, the final analysis was conducted with 150 participants, including 75 women in the intervention group and 75 women in the control group. Participants were allocated to the intervention and control groups using a non-randomized systematic recruitment procedure based on the order of application to the Family Health Center. Women who met the eligibility criteria and agreed to participate were assigned sequentially according to the study plan. To minimize potential contamination between groups, data collection for the intervention and control groups was conducted at different times. Baseline comparability between groups was subsequently assessed using chi-square tests and an independent samples *t*-test, and no statistically significant differences were identified in sociodemographic characteristics.

### 2.4. Sample Size Calculation

To determine the adequacy of the sample size, an a priori power analysis was performed using G*Power 3.1, a statistical software package designed for power and sample size calculations [14]. The independent samples *t*-test was used as the basis for the power analysis, with a moderate effect size (d = 0.50), a significance level of α = 0.05, and statistical power (1 − β) = 0.80. The analysis indicated that a minimum sample size of 128 participants was required. Considering the possibility of participant attrition, 154 women were initially enrolled in the study. Following the loss of four participants during follow-up, the final analysis was conducted with 150 participants. Therefore, the final sample size exceeded the minimum required sample size and was considered sufficient to ensure adequate statistical power and support the reliability of the study findings.

Inclusion criteria for the study were: women aged 18–49 who applied to a Family Health Center, were parents of adolescent girls (ages 10–18), could read and write in Turkish, voluntarily agreed to participate in the study, and provided written informed consent. Exclusion criteria: individuals who had previously received the HPV vaccine, those diagnosed with cervical cancer, and those with cognitive or psychiatric problems that hindered communication were excluded from the study.

### 2.5. Data Collection Tools

Research data were collected using the “Demographic Information Form”, “Cervical Cancer Knowledge Scale (CCKS)” and “Carolina HPV Immunization Attitudes and Beliefs Scale (CHIAS)”.

**Demographic Information Form:** The form, prepared by the researchers in line with the literature, consists of a total of 10 questions regarding age, marital status, education level, employment status, income level, number of children, Pap smear test status, information about HPV status, and perception of cervical cancer risk. The Participant Information Form was developed by researchers following a review of the relevant literature and was reviewed by 6 experts in public health nursing and women’s health to ensure content appropriateness and clarity.

**Cervical Cancer Knowledge Scale (CCKS):** The CCKS was used to assess participants’ knowledge levels regarding cervical cancer risk factors, symptoms, and prevention methods. The scale was developed by Haward et al. (2022) [15], and its Turkish validity and reliability study was conducted by Ergöz Aksoy and Bilgiç (2024) [16].

The scale consists of eight items in total, and the items are answered on a three-point Likert scale as “True,” “False,” and “I do not know.” Seven items are scored directly, while one item is reverse scored. Correct responses are scored as 1 point, whereas incorrect and “I do not know” responses are scored as 0 points. Higher scores obtained from the scale indicate a higher level of cervical cancer knowledge. The Cronbach’s alpha coefficient of the Turkish version of the scale was reported as 0.80. In the present study, the Cronbach’s alpha coefficient of the CCKS was found to be 0.709.

**Carolina HPV Immunization Attitudes and Beliefs Scale (CHIAS):** The scale, originally developed by McRee et al. (2010) [17], was adapted into Turkish by Sunar and Kahyaoğlu Süt (2019) [18]. The scale consists of 16 items and four factors.

The first factor, “Harms,” includes six items related to the perceived potential harms of the vaccine, including health concerns and the possibility that vaccination may increase girls’ likelihood of becoming sexually active. The second factor, “Barriers,” consists of five items assessing perceived barriers to HPV vaccination, including cost and access to a healthcare provider. The third factor, “Effectiveness,” includes two items related to the perceived effectiveness of the HPV vaccine in preventing genital warts and cervical cancer. The fourth factor, “Uncertainty,” consists of three items evaluating perceptions regarding adequate knowledge about the HPV vaccine and social vaccination norms.

The item order in the original scale and the version used in this study was identical. The first factor, “Harms,” includes items 1–6 and uses a four-point Likert scale (1 = Strongly disagree, 2 = Partially disagree, 3 = Partially agree, 4 = Strongly agree). The second factor, “Barriers,” includes items 7–11 and uses a three-point Likert scale (1 = Not difficult at all, 2 = Somewhat difficult, 3 = Very difficult). The third factor, “Effectiveness,” includes items 12–13 and uses a four-point Likert scale (1 = Very ineffective, 2 = Moderately effective, 3 = Very effective, 4 = Extremely effective). The fourth factor, “Uncertainty,” includes items 14–16 and uses a four-point Likert scale (1 = Strongly disagree, 2 = Partially disagree, 3 = Partially agree, 4 = Strongly agree).

In the original scale, the possible score range for all factor scores was between 1.0 and 4.0. Acceptable internal consistency coefficients were reported for all factors: Harms α = 0.69, Barriers α = 0.69, Effectiveness α = 0.61, and Uncertainty α = 0.66. CHIAS scores were interpreted according to the scoring procedure of the instrument. Negatively worded items in the CHIAS were reverse coded during data entry and analysis in SPSS according to the scoring procedure of the instrument. Accordingly, higher scores on the Harms, Barriers, and Effectiveness subscales indicated more favorable HPV vaccination attitudes and beliefs, whereas lower scores on the Uncertainty subscale indicated reduced uncertainty regarding HPV vaccination. In the Barriers subdimension of the CHIAS, higher scores indicate lower perceived barriers toward HPV vaccination and more positive perceptions regarding vaccine accessibility and applicability. The Cronbach’s alpha coefficient of the original scale was reported as 0.62. In the present study, Cronbach’s alpha coefficient of the scale was found to be 0.91.

### 2.6. Data Collection Process

The data collection process was initiated after obtaining the necessary ethical committee approval and institutional permissions required for conducting the study. Potential participants were approached consecutively while attending the Family Health Center for routine healthcare services. Eligibility was assessed according to the predefined inclusion and exclusion criteria. Women who met the eligibility criteria and agreed to participate provided written informed consent before enrollment. All collected data were kept confidential, stored in password-protected electronic files, and used solely for research purposes. No financial compensation, gifts, or material incentives were provided to participants for participation in the study.

### 2.7. Intervention Group Procedure

During the initial interview, pretest data were collected from participants in the intervention group using the Descriptive Information Form, the Cervical Cancer Knowledge Scale (CCKS), and the Carolina HPV Immunization Attitudes and Beliefs Scale (CHIAS).

The educational intervention was delivered over a two-week period and consisted of two face-to-face educational sessions. Each session lasted approximately 30–40 min and was conducted in small groups of four to five participants in a private counseling room within the Family Health Center.

The educational content was developed by the researchers based on the core constructs of the Health Belief Model, including perceived susceptibility, perceived severity, perceived benefits, and perceived barriers.

The first educational session focused on cervical cancer, including its definition, risk factors, potential consequences, perceived susceptibility, perceived severity, and the importance and benefits of cervical cancer screening. The second educational session focused on HPV infection and transmission routes, HPV vaccine effectiveness and safety, correction of common misconceptions regarding HPV vaccination, and discussion of perceived barriers related to vaccine acceptance.

Interactive discussions, question-and-answer techniques, and real-life examples were used throughout both sessions to reinforce learning and encourage participant engagement. Educational materials were prepared by the researchers based on current evidence regarding cervical cancer prevention, HPV infection, HPV vaccination, and the Health Belief Model framework. The educational sessions were delivered by researchers with experience in women’s health, health education, and primary healthcare practice. To ensure intervention standardization, all educational sessions followed the same structured content, sequence of topics, educational materials, and delivery procedures. The sessions were conducted using a predefined educational protocol developed by the research team. The same educators delivered all sessions throughout the study period to maintain consistency in intervention implementation.
**HBM Construct****Educational Content Applied to the Intervention Group****Perceived Susceptibility** ────────────────────Information was provided on the prevalence of cervical cancer and HPV infection, risk factors, transmission routes, and their effects on women’s health to increase perceived susceptibility.**Perceived Severity** ───────────────Information was provided on the physical, psychological, social, and economic consequences of delayed diagnosis to increase awareness of disease severity.**Perceived Benefits** ────────────────The benefits of HPV vaccination, regular gynecological examinations, and screening programs for cervical cancer prevention were emphasized.**Perceived Barriers** ───────────────Barriers to HPV vaccination and cervical cancer screening, including fear, lack of knowledge, cost, embarrassment, and misconceptions, were discussed to reduce these obstacles.**Health Motivation** ──────────────Education and counseling were provided to encourage HPV vaccination, participation in screening programs, and maintenance of preventive health behaviors.**Self-Efficacy** ──────────The program aimed to strengthen women’s confidence in adopting preventive behaviors, accessing screening services, and making informed health decisions.

At the end of each session, participants were encouraged to ask questions and discuss any concerns regarding cervical cancer prevention and HPV vaccination. The educational materials used in the intervention are available from the corresponding author upon reasonable request.

The posttest assessment was conducted 15 days after completion of the educational program using the same data collection instruments.

### 2.8. Control Group

Pretest data were collected from participants in the control group during the initial interview by administering the Demographic Information Form, the CCKS, and the CHIAS.

No educational interventions were applied to the control group other than routine Family Health Center services. The posttest was administered at the same time as the intervention group, and the same data collection tools were used.

Data collection processes were carried out at different times to minimize information sharing and potential interaction between the groups.

Participants in the control group received routine Family Health Center services, including general health assessments, maternal and child health services, vaccination counseling routinely provided within national healthcare programs, and other standard primary healthcare services when indicated. No structured education, counseling session, educational materials, or information specifically related to cervical cancer, HPV infection, HPV vaccination, or the Health Belief Model was provided during the study period. To avoid potential contamination, participants in the control group did not have access to the educational materials used in the intervention until completion of the posttest assessments.

### 2.9. Data Analysis

The data obtained from the research were analyzed using the IBM SPSS Statistics 25 software package. Cronbach’s Alpha coefficients were calculated to determine the reliability levels of the scales. The suitability of the data to a normal distribution was evaluated by examining the skewness and kurtosis values. Since the skewness and kurtosis values were in the range of ±3, it was accepted that the data showed a normal distribution, and it was decided to use parametric tests [19]. Number, percentage, mean, and standard deviation values were used to evaluate the sociodemographic characteristics of the participants. Dependent samples *t*-test was used for within-group pretest–posttest comparisons of the intervention and control groups, and independent samples *t*-test was used for between-group comparisons. In addition, mixed-design analysis of variance (ANOVA) was performed to evaluate Group × Time interaction effects, and partial eta squared (η^2^p) values were calculated as measures of effect size. The statistical significance level was accepted as *p* < 0.05. Prior to analysis, the dataset was examined for missing values and data entry errors. No missing data was identified in the final dataset; therefore, no imputation procedures were required.

### 2.10. Ethical Aspects

Ethical approval was obtained from the Muş Alparslan University Scientific Research and Publication Ethics Board to conduct the research (Decision No: 238631, Date: 9 March 2026). In addition, the necessary institutional permissions were obtained from the institution where the research was conducted. Before participating in the research, all participants were given detailed information about the study, and written informed consent was obtained from individuals who voluntarily agreed to participate. Participants were informed that they could withdraw from the research at any time. The research was conducted in accordance with the principles of the Helsinki Declaration.

## 3. Results

Table 1 shows that the participants in the control and intervention groups have similar sociodemographic distributions. In both groups, the vast majority of participants are married (control: 90.7%; intervention: 89.3%). In terms of educational status, the highest percentage in both groups consists of high school graduates (control: 48.0%; intervention: 50.7%). It was determined that the majority of participants are unemployed (81.3% for both groups).

When income status was examined, it was found that the majority of participants had an income lower than their expenses (control: 58.7%; intervention: 61.3%). Regarding the number of children, the most common categories in both groups were having two children, four children, and five or more children.

It was determined that most participants had never undergone a Pap smear test (control: 76.0%; intervention: 74.7%) and had not received information about the HPV vaccine (control: 68.0%; intervention: 69.3%). In addition, a considerable proportion of participants reported that they did not perceive themselves to be at risk for cervical cancer (control: 64.0%; intervention: 61.3%). In both groups, the proportion of participants without a family history of cervical cancer was higher (control: 76.0%; intervention: 74.7%).

The mean age of participants was 42.33 ± 5.46 years in the control group and 42.25 ± 5.29 years in the intervention group. Overall, it can be concluded that the control and intervention groups were similar in terms of sociodemographic characteristics (Table 1).

When Table 2 is examined, it is observed that there are notable differences in mean scores between pretest and posttest measurements. The mean pretest cervical cancer knowledge score was 2.13 ± 0.78, which increased to 4.67 ± 2.71 in the posttest. Similarly, the mean score of the CHIAS was 24.19 ± 5.27 at pretest and increased to 32.73 ± 9.34 at posttest.

For the subdimensions, the mean score of the “Harms” subscale increased from 9.47 ± 2.08 to 14.74 ± 5.50, the “Barriers” subscale increased from 5.73 ± 1.80 to 8.12 ± 2.76, and the “Effectiveness” subscale increased from 3.23 ± 1.30 to 5.23 ± 2.28. In contrast, the mean score of the “Uncertainty” subscale decreased from 5.76 ± 0.44 at pretest to 4.64 ± 1.34 at posttest. These descriptive findings suggest improvements in knowledge and CHIAS-related scores between pretest and posttest measurements.

Regarding the assumption of normality, skewness and kurtosis values for all variables were within ±3. Accordingly, the data were considered to be normally distributed, and the use of parametric tests was deemed appropriate. In particular, skewness values ranged between −0.994 and 0.611, while kurtosis values ranged between −2.007 and 0.309, indicating an acceptable level of normal distribution.

In addition, Cronbach’s alpha coefficients indicating the reliability of the scales ranged between 0.709 and 0.942. These values indicate that the measurement tools and their subdimensions demonstrated acceptable to high internal consistency, confirming the reliability of the measurements.

Table 3 shows that there were significant differences in CHIAS and their subdimensions between pretest and posttest comparisons in the control and intervention groups.

In the control group, no statistically significant differences were found between pretest and posttest scores for the total CHIAS or for the subdimensions of harms, barriers, effectiveness, and uncertainty (*p* > 0.05). In contrast, in the intervention group, statistically significant differences were observed between pretest and posttest scores for all variables (*p* < 0.001).

In the intervention group, the mean total CHIAS score increased from 24.32 ± 5.30 to 41.23 ± 2.51. Similarly, the mean score for the “Harms” subscale increased from 9.53 ± 2.07 to 19.99 ± 1.24, the “Barriers” subscale from 5.77 ± 1.80 to 10.48 ± 1.12, and the “Effectiveness” subscale from 3.27 ± 1.31 to 7.24 ± 0.84. In contrast, the mean score for the “Uncertainty” subscale decreased from 5.75 ± 0.44 to 3.52 ± 0.86. These findings indicate that the HBM–based education improved HPV vaccination attitudes and beliefs and reduced uncertainty in the intervention group.

Between-group comparisons revealed no statistically significant differences between the control and intervention groups at pretest for any of the variables (*p* > 0.05), indicating baseline homogeneity between groups. However, at posttest, statistically significant differences were found between the groups in the total CHIAS score and all subdimensions (*p* < 0.001). At posttest, the intervention group showed higher mean scores in the total scale and in the harms, barriers, and effectiveness subdimensions, while lower uncertainty scores compared with the control group. These findings suggest a positive association with the HBM–based education program in the intervention group. Effect size analyses indicated very large intervention effects across all HPV vaccination attitude and belief dimensions, with Cohen’s d values ranging from 2.05 to 5.10.

Mixed-design ANOVA revealed significant Group × Time interaction effects for total CHIAS scores (F(1,148) = 960.275, *p* < 0.001, η^2^p = 0.866), Harms (F(1,148) = 1726.940, *p* < 0.001, η^2^p = 0.921), Barriers (F(1,148) = 325.430, *p* < 0.001, η^2^p = 0.687), Effectiveness (F(1,148) = 721.039, *p* < 0.001, η^2^p = 0.830), and Uncertainty (F(1,148) = 282.822, *p* < 0.001, η^2^p = 0.656), indicating that changes over time differed significantly between the intervention and control groups.

Table 4 shows differences in CCKS pretest–posttest scores between the control and intervention groups. In the control group, no statistically significant difference was found between pretest and posttest knowledge scores (*p* > 0.05). In contrast, a statistically highly significant difference was observed between pretest and posttest scores in the intervention group (*p* < 0.001).

In the intervention group, the mean cervical cancer knowledge score increased from 2.13 ± 0.79 to 7.24 ± 0.84. In the control group, the mean pretest score was 2.13 ± 0.78 and the mean posttest score was 2.09 ± 0.84, indicating no significant change. These findings suggest that the educational intervention was associated with higher cervical cancer knowledge scores.

Between-group comparisons showed no statistically significant difference in pretest knowledge scores between the control and intervention groups (t = 0.000; *p* = 1.000), indicating baseline equivalence. However, a statistically significant difference was found between the groups at posttest (t = −37.592; *p* < 0.001), with the intervention group showing substantially higher knowledge scores than the control group. These results indicate that the HBM–based education program was associated with higher cervical cancer knowledge among women.

A significant Group × Time interaction effect was also observed for cervical cancer knowledge scores (F(1,148) = 1096.867, *p* < 0.001, η^2^p = 0.881), indicating substantially greater improvement in the intervention group compared with the control group.

## 4. Discussion

The findings of this study, conducted to evaluate the effect of HBM-based education program on women’s cervical cancer knowledge and their attitudes and beliefs towards the HPV vaccine, are discussed in light of the existing literature. A notable finding was that most participants were unemployed and approximately three-quarters had never undergone a Pap smear test despite their mean age being approximately 42 years. This finding is important in light of WHO cervical cancer screening recommendations, which emphasize regular screening from age 30 in the general population. The low screening history observed in this study may reflect limited awareness of screening recommendations, low perceived susceptibility to cervical cancer, sociocultural barriers, and insufficient preventive health literacy. Therefore, women attending Family Health Centers may represent an important target group for structured cervical cancer and HPV-related education.

In this study, it was determined that the attitudes and beliefs of women in the intervention group regarding cervical cancer and the HPV vaccine increased significantly after the HBM-based education program. The significant increase in cervical cancer knowledge scores and attitude and belief scores regarding the HPV vaccine in the intervention group in the posttest indicates that the education increased women’s awareness of cervical cancer, HPV infection, and prevention methods. This may be related to the participants’ low initial knowledge levels and the highly structured nature of the education content. The highly structured nature of the intervention refers to the systematic organization of educational content according to the core constructs of the Health Belief Model. The program addressed perceived susceptibility, perceived severity, perceived benefits, perceived barriers, health motivation, and self-efficacy through standardized educational messages, interactive discussion, question-and-answer methods, and practical examples related to cervical cancer prevention and HPV vaccination. In contrast, no significant change was observed between the pretest and posttest scores in the control group. This finding supports hypothesis H1. The literature also reports that HBM-based education programs are effective in increasing knowledge levels about cervical cancer and HPV [20,21,22,23]. It is particularly noted that structured education programs strengthen women’s risk perception regarding the disease, increase awareness of early detection methods, and support preventive health behaviors [24,25]. From a Health Belief Model perspective, increased knowledge regarding cervical cancer may contribute to greater perceived susceptibility and perceived severity, which are important precursors of preventive health behaviors. Improved awareness of cervical cancer risk factors, disease consequences, and screening benefits may therefore enhance women’s motivation to participate in preventive health practices, including cervical cancer screening and HPV-related preventive behaviors. The magnitude of improvement observed in the present study may be partly explained by the relatively low baseline levels of cervical cancer and HPV-related knowledge among participants. A substantial proportion of women reported that they had never received information about HPV vaccination and had never undergone cervical cancer screening. Consequently, even a brief educational intervention may have produced a more pronounced effect than would be expected in populations with higher baseline awareness. In addition, the educational sessions were delivered within a trusted primary healthcare setting, which may have facilitated participant engagement and receptiveness to health information.

In the study, it was found that attitude and belief scores towards the HPV vaccine increased significantly in the intervention group after the education. This result supports hypothesis H2. Many studies have shown that educational interventions are effective in developing positive attitudes towards the HPV vaccine [22,26,27]. In particular, it is reported that information given about the safety, effectiveness, and role of the HPV vaccine in protecting against cervical cancer increases individuals’ acceptance of the vaccine [28]. The inclusion of explanations about the protective effect, safety, and misconceptions of the HPV vaccine in the education program applied in the study may have contributed to the development of positive attitudes towards the HPV vaccine in women. In addition, the fact that a large proportion of the participants had not previously received information about the HPV vaccine may have caused the change obtained after the education to become more pronounced. The observed changes in HPV vaccination attitudes and beliefs can also be interpreted within the framework of the Health Belief Model. The increase in effectiveness scores suggests stronger perceived benefits regarding HPV vaccination, whereas the reduction in uncertainty reflects improved confidence and health motivation related to vaccine decision-making. These findings indicate that the educational intervention may have positively influenced several cognitive determinants targeted by the Health Belief Model, thereby supporting more favorable attitudes toward HPV vaccination. The findings may also have implications for the scalability of similar interventions in primary healthcare settings. Family Health Centers routinely interact with women across different stages of life and therefore provide a practical platform for delivering structured educational programs. Given the relatively low resource requirements of the intervention, similar HBM-based educational approaches may be feasible in other primary care settings where HPV vaccine awareness and cervical cancer screening participation remain suboptimal.

The short-term improvements observed in the present study are consistent with previous Health Belief Model–based educational interventions targeting cervical cancer prevention and HPV-related health behaviors. Previous studies have reported that educational interventions grounded in HBM can improve cervical cancer knowledge, screening intentions, and HPV-related attitudes beyond the immediate post-intervention period, with some benefits persisting for several months after program completion [20,21,24]. Systematic reviews have also suggested that theory-based educational and counseling interventions may contribute to sustained improvements in preventive health beliefs and vaccine-related decision-making [24,27]. Although the present study demonstrated significant improvements after a 15-day follow-up period, longer-term assessments are needed to determine whether these positive changes are maintained over time.

Despite the positive findings, the short follow-up period should be considered when interpreting the results. The observed changes may partly reflect short-term recall of educational content rather than stable and sustained changes in attitudes and beliefs. While short follow-up assessments are useful for evaluating immediate educational outcomes, longer follow-up periods are necessary to determine whether these improvements are maintained over time and translate into lasting behavioral changes related to HPV vaccination and cervical cancer prevention.

Beyond the overall improvement in HPV vaccination attitudes and beliefs, changes in specific HBM-related dimensions also warrant consideration. In this study, it was determined that the perceived barrier scores towards the HPV vaccine changed positively in the intervention group after education. This finding directly corresponds to the perceived barriers construct of the Health Belief Model, which proposes that individuals are more likely to adopt preventive health behaviors when perceived obstacles are reduced. This finding supports the positive effect of HBM-based education programs on attitudes and beliefs towards the HPV vaccine. According to HBM, perceived barriers are one of the key factors affecting an individual’s ability to engage in preventive health behavior. It is known that factors such as cost, safety concerns, lack of information, and misconceptions regarding the HPV vaccine negatively affect vaccine acceptance [26,29]. In this study, correcting misconceptions, providing information about the safety of the HPV vaccine, and emphasizing the benefits of the vaccine during the education process may have reduced the perceived barrier levels of women. Similarly, current systematic reviews report that education and counseling-based interventions are effective in reducing hesitation and perceived barriers towards the HPV vaccine [27,30].

Another notable finding in the study is the significant decrease in the uncertainty subscale scores in the intervention group after the training. This result indicates that HBM-based education programs can positively influence women’s health behaviors by reducing the level of uncertainty regarding the HPV vaccine. The decrease in uncertainty is thought to be related to the increased knowledge of participants regarding HPV infection, the HPV vaccine, and vaccination norms. The literature states that a lack of information increases the level of hesitation and uncertainty regarding the HPV vaccine, and that educational interventions contribute to individuals developing more positive health behaviors by reducing this uncertainty [22,28,31]. It is stated that trainings based on the perceived benefits and perceived barriers dimensions of HBM, in particular, positively influence individuals’ health decisions [21,24].

The observed improvements in knowledge and HPV vaccination attitudes may reflect changes in key Health Belief Model constructs, including perceived susceptibility, perceived severity, perceived benefits, and perceived barriers, suggesting that theory-based educational interventions can facilitate positive health-related perceptions and decision-making.

The significant Group × Time interaction effects observed across all outcome measures further strengthen the interpretation of the findings. These interaction effects indicate that the improvements observed in cervical cancer knowledge and HPV vaccination attitudes were not merely attributable to the passage of time but were specifically associated with participation in the HBM-based educational intervention.

Overall, the findings indicate that structured education based on the Health Belief Model implemented in Family Health Centers was associated with higher levels of cervical cancer and HPV knowledge, more favorable attitudes toward HPV vaccination, lower perceived barriers to vaccination, and reduced uncertainty regarding HPV vaccination. These findings suggest that structured education initiatives implemented in primary healthcare settings can contribute to improving women’s health, raising awareness of cervical cancer, and promoting HPV vaccine acceptance.

### Limitations of the Study

Several limitations should be considered when interpreting the findings of this study. First, the quasi-experimental non-randomized design may have introduced selection bias and limits the ability to draw causal inferences. Although the intervention and control groups were comparable on baseline characteristics, potential threats to internal validity cannot be completely excluded. Second, the posttest assessment was conducted shortly after completion of the intervention, and no 3-month or 6-month follow-up assessment was performed; therefore, the long-term sustainability of the observed improvements in cervical cancer knowledge and HPV vaccination attitudes could not be determined. Third, all outcome measures were based on self-reported responses and may therefore be subject to recall bias or social desirability bias. Furthermore, the study was conducted in a single Family Health Center, which may limit the generalizability of the findings to other populations and healthcare settings. Finally, although the intervention was associated with improvements in cervical cancer knowledge and HPV vaccination attitudes, actual behavioral outcomes such as HPV vaccine uptake or cervical cancer screening participation were not assessed. Future studies should incorporate longer follow-up periods, multi-center designs, and objective behavioral outcome measures.

## 5. Conclusions

This study demonstrated that a Health Belief Model (HBM)-based education program implemented in Family Health Centers was associated with improved cervical cancer and HPV knowledge, more favorable HPV vaccination attitudes and beliefs, reduced perceived barriers, and lower uncertainty regarding HPV vaccination. Significant Group × Time interaction effects observed across all outcome measures further support the positive association between the educational intervention and improvements in cervical cancer knowledge and HPV vaccination-related attitudes and beliefs.

The findings suggest that theory-based educational interventions delivered in primary healthcare settings may contribute to strengthening preventive health behaviors and improving awareness of cervical cancer prevention.

### Practical Implications and Recommendations

These findings are particularly important in settings where HPV vaccination awareness remains limited and participation in cervical cancer screening programs is suboptimal. The study demonstrates that relatively brief, theory-based educational interventions delivered within routine primary healthcare services may positively influence key cognitive determinants of preventive health behavior. Therefore, strengthening educational activities in Family Health Centers may contribute to broader public health efforts aimed at reducing the burden of cervical cancer. Family Health Centers provide an accessible and trusted environment for delivering educational programs aimed at increasing HPV-related knowledge and promoting positive vaccination attitudes among women.

Based on the findings of this study, the following recommendations are proposed:

1. Structured HBM-based cervical cancer and HPV education programs should be integrated into routine preventive healthcare services provided by Family Health Centers.
2. Primary healthcare professionals, particularly nurses and family physicians, should receive regular training on HPV vaccination counseling and evidence-based communication strategies.
3. Educational materials addressing common misconceptions, safety concerns, and perceived barriers related to HPV vaccination should be routinely distributed in primary care settings.
4. Community-based awareness campaigns should be developed to improve women’s knowledge of cervical cancer prevention, HPV infection, and HPV vaccination.
5. Digital health education approaches, including mobile applications and online educational platforms, should be incorporated into cervical cancer and HPV awareness initiatives to expand access to reliable health information.
6. Policymakers should consider strengthening national awareness initiatives and improving access to HPV vaccination services, particularly among underserved populations.
7. Healthcare institutions should monitor the effectiveness of educational interventions by assessing changes in cervical cancer screening participation and HPV vaccination uptake.
8. Future multicenter studies should evaluate long-term behavioral outcomes, cost-effectiveness, and scalability of HBM-based educational interventions across diverse primary healthcare settings.
9. Collaboration between Family Health Centers, schools, and community organizations should be strengthened to increase public awareness of HPV infection, cervical cancer prevention, and HPV vaccination.
10. National cervical cancer prevention strategies should incorporate theory-based educational interventions as a routine component of women’s preventive health services and health promotion programs.

Overall, the findings suggest that integrating structured HBM-based educational interventions into routine primary healthcare services may represent a practical and scalable strategy for improving cervical cancer prevention efforts, strengthening HPV vaccine acceptance, and supporting public health goals aimed at reducing the burden of cervical cancer.

## Figures and Tables

**Table 1:** Distribution of Participants’ Sociodemographic Characteristics by Groups.

Variable	Category	Control n (%)	Intervention n (%)	χ^2^ (df)	*p*
**Marital Status**	Married	68 (90.7%)	67 (89.3%)	0.074 (1)	0.785
	Single	7 (9.3%)	8 (10.7%)		
**Education Level**	Primary education	14 (18.7%)	16 (21.3%)	0.535 (3)	0.911
	High school	36 (48.0%)	38 (50.7%)		
	Undergraduate	19 (25.3%)	16 (21.3%)		
	Postgraduate	6 (8.0%)	5 (6.7%)		
**Employment Status**	Employed	14 (18.7%)	14 (18.7%)	0.000 (1)	1.000
	Unemployed	61 (81.3%)	61 (81.3%)		
**Children**	1	4 (5.3%)	4 (5.3%)	0.117 (4)	0.998
	2	22 (29.3%)	21 (28.0%)		
	3	13 (17.3%)	12 (16.0%)		
	4	18 (24.0%)	19 (25.3%)		
	5 and above	18 (24.0%)	19 (25.3%)		
**Pap Smear History**	Yes	18 (24.0%)	19 (25.3%)	0.036 (1)	0.850
	No	57 (76.0%)	56 (74.7%)		
**HPV Vaccine Information**	Yes	24 (32.0%)	23 (30.7%)	0.31 (1)	0.860
	No	51 (68.0%)	52 (69.3%)		
**Perceived Risk of Cervical Cancer**	Yes	18 (24.0%)	19 (25.3%)	0.122 (2)	0.941
	No	48 (64.0%)	46 (61.3%)		
	Undecided	9 (12.0%)	10 (13.3%)		
**Family History of Cervical Cancer**	Yes	18 (24.0%)	19 (25.3%)	0.036 (1)	0.850
	No	57 (76.0%)	56 (74.7%)		
Income Status	Income less than expenses	44 (58.7%)	46 (61.3%)	0.323 (2)	0.851
	Income equal to expenses	21 (28.0%)	18 (24.0%)		
	Income more than expenses	10 (13.3%)	11 (14.7%)		
Age	Mean ± SD	42.33 ± 5.46	42.25 ± 5.29	t = 0.091	0.928

Note: *p* values were calculated using Chi-square tests for categorical variables and an independent samples *t*-test for age.

**Table 2:** Descriptive Statistics and Normality Values of Participants in the Control and Intervention Groups.

Variable	n	Min	Max	Mean	SD	Skewness	Kurtosis	Cronbach’s Alpha
Pretest Cervical Cancer Knowledge	150	1.00	4.00	2.13	0.783	0.611	0.309	0.709
Posttest Cervical Cancer Knowledge	150	0.00	8.00	4.67	2.713	−0.007	−1.694	0.862
Pretest CHIAS	150	19.00	30.00	24.19	5.273	0.040	−2.007	0.914
Posttest CHIAS	150	18.00	43.00	32.73	9.340	−0.268	−1.496	0.894
*Pretest Harms*	150	7.00	12.00	9.47	2.075	0.025	−1.800	0.815
*Posttest Harms*	150	6.00	21.00	14.74	5.499	−0.103	−1.718	0.887
*Pretest Barriers*	150	4.00	8.00	5.73	1.800	0.142	−1.865	0.942
*Posttest Barriers*	150	4.00	12.00	8.12	2.761	−0.239	−1.255	0.927
*Pretest Effectiveness*	150	2.00	5.00	3.23	1.303	0.240	−1.729	0.890
*Posttest Effectiveness*	150	2.00	8.00	5.23	2.280	−0.213	−1.430	0.939
*Pretest Uncertainty*	150	5.00	7.00	5.76	0.444	−0.994	−0.348	0.752
*Posttest Uncertainty*	150	2.00	7.00	4.64	1.337	−0.492	−0.531	0.788

**Table 3:** Independent and Paired Samples *t*-Test Results for HPV Vaccination Attitudes and Beliefs Scale (Pretest–Posttest and Between-Group Comparisons).

Variable	Group	Pretest Mean ± SD	Posttest Mean ± SD	Within-Group t	*p*	Cohen’s d
**CHIAS**	Control	24.05 ± 5.28	24.24 ± 4.81	−1.669	0.099	
	Intervention	24.32 ± 5.30	41.23 ± 2.51	−32.030	<0.001 *	3.70
	**Between-group**	t: −0.309 *p*: 0.758	t: −27.123 *p*: <0.001 *			
	Group × Time Interaction			F(1,148) = 960.275	<0.001; η^2^p = 0.866	
***Harms***	Control	9.40 ± 2.09	9.49 ± 1.89	−1.186	0.239	
	Intervention	9.53 ± 2.07	19.99 ± 1.24	−44.189	<0.001 *	5.10
	**Between-group**	t: −0.392 *p*: 0.695	t: −40.222 *p*: <0.001 *			
	Group × Time Interaction			F(1,148) = 1726.940	<0.001; η^2^p = 0.921	
***Barriers***	Control	5.68 ± 1.79	5.76 ± 1.68	−1.349	0.181	
	Intervention	5.77 ± 1.80	10.48 ± 1.12	−18.863	<0.001 *	2.18
	**Between-group**	t: −0.318 *p*: 0.751	t: −20.288 *p*: <0.001 *			
	Group × Time Interaction			F(1,148) = 325.430	<0.001; η^2^p = 0.687	
***Effectiveness***	Control	3.20 ± 1.30	3.23 ± 1.27	−0.630	0.531	
	Intervention	3.27 ± 1.31	7.24 ± 0.84	−28.230	<0.001 *	3.26
	**Between-group**	t: −0.312 *p*: 0.755	t: −22.876 *p*: <0.001 *			
	Group × Time Interaction			F(1,148) = 721.039	<0.001; η^2^p = 0.830	
***Uncertainty***	Control	5.77 ± 0.45	5.76 ± 0.57	0.331	0.741	
	Intervention	5.75 ± 0.44	3.52 ± 0.86	17.770	<0.001 *	2.05
	**Between-group**	t: 0.367 *p*: 0.714	t: 18.847 *p*: <0.001 *			
	Group× Time Interaction			F(1,148) = 282.822	<0.001; η^2^p = 0.656	

* *p* < 0.05 indicates statistical significance. Cohen’s d values were calculated for within-group pretest–posttest changes in the intervention group using the paired-samples effect size formula (dz = t/√n). Values of 0.20, 0.50, and 0.80 indicate small, medium, and large effect sizes, respectively.

**Table 4:** Independent and Paired Samples *t*-Test Results for Cervical Cancer Knowledge Scale (Pretest–Posttest and Between-Group Comparisons).

Variable	Group	Pretest Mean ± SD	Posttest Mean ± SD	Within-Group t	*p*	Cohen’s d
**Cervical Cancer Knowledge**	Control	2.13 ± 0.78	2.09 ± 0.84	0.505	0.615	
	Intervention	2.13 ± 0.79	7.24 ± 0.84	−38.207	<0.001 *	4.41
	**Between-group**	t: 0.000 *p*: 1.000	t: −37.592 *p*: <0.001 *			
	Group × Time Interaction			F(1,148) = 1096.867	<0.001; η^2^p = 0.881	

* *p* < 0.05 indicates statistical significance. Cohen’s d values were calculated for within-group pretest–posttest changes in the intervention group using the paired-samples effect size formula (dz = t/√n).

## Data Availability

The data presented in this study are available from the corresponding author upon reasonable request, because the dataset consists of individual-level responses collected from human participants. Although the dataset has been anonymized, it is not publicly available in order to protect participant confidentiality and privacy. Therefore, the data are available from the corresponding author upon reasonable request for legitimate scientific research purposes.

## Abbreviations

HBM	Health Belief Model
HPV	Human Papillomavirus
CHIAS	Carolina HPV Immunization Attitudes and Beliefs Scale
CCKS	Cervical Cancer Knowledge Scale

## References

[1] World Health Organization (2022). Cervical Cancer. https://www.who.int/news-room/fact-sheets/detail/cervical-cancer.

[2] Arbyn M., Weiderpass E., Bruni L., de Sanjosé S., Saraiya M., Ferlay J., Bray F. (2020). Estimates of incidence and mortality of cervical cancer in 2018. Lancet Glob. Health.

[3] Walboomers J.M., Jacobs M.V., Manos M.M., Bosch F.X., Kummer J.A., Shah K.V., Snijders P.J., Peto J., Meijer C.J., Muñoz N. (1999). Human papillomavirus is a necessary cause of invasive cervical cancer worldwide. J. Pathol..

[4] Lei J., Ploner A., Elfström K.M., Wang J., Roth A., Fang F., Sundström K., Dillner J., Sparén P. (2020). HPV vaccination and the risk of invasive cervical cancer. N. Engl. J. Med..

[5] Bruni L., Saura-Lázaro A., Montoliu A., Brotons M., Alemany L., Diallo M.S., Afsar O.Z., LaMontagne D.S., Mosina L., Contreras M. (2021). HPV vaccination introduction worldwide and WHO and UNICEF estimates of national HPV immunization coverage 2010-2019. Prev. Med..

[6] Holman D.M., Benard V., Roland K.B., Watson M., Liddon N., Stokley S. (2014). Barriers to human papillomavirus vaccination among US adolescents. JAMA Pediatr..

[7] Kessels S.J.M., Marshall H.S., Watson M., Braunack-Mayer A.J., Reuzel R., Tooher R.L. (2012). Factors associated with HPV vaccine uptake. Vaccine.

[8] Arbyn M., Smith S.B., Temin S., Sultana F., Castle P. (2018). Detecting cervical precancer and reaching underserved women. CA A Cancer J. Clin..

[9] Gultekin M., Zayifoglu Karaca M., Kucukyildiz I., Dundar S., Boztas G., Semra Turan H., Hacikamiloglu E., Murtuza K., Keskinkilic B., Sencan I. (2018). Initial results of population based cervical cancer screening program using HPV testing in one million Turkish women. Int. J. Cancer.

[10] Nutbeam D. (2008). The evolving concept of health literacy. Soc. Sci. Med..

[11] Rosenstock I.M. (1974). Historical origins of the Health Belief Model. Health Educ. Monogr..

[12] Brewer N.T., Chapman G.B., Gibbons F.X., Gerrard M., McCaul K.D., Weinstein N.D. (2007). Meta-analysis of the relationship between risk perception and health behavior: The example of vaccination. Health Psychol. Off. J. Div. Health Psychol..

[13] Kim H.W. (2012). Knowledge about human papillomavirus (HPV), and health beliefs and intention to recommend HPV vaccination. Vaccine.

[14] Kang H. (2021). Sample size determination and power analysis using the G*Power software. J. Educ. Eval. Health Prof..

[15] Haward B., Tatar O., Zhu P., Griffin-Mathieu G., Perez S., Shapiro G.K., McBride E., Zimet G.D., Rosberger Z. (2022). Development and validation of the cervical cancer knowledge scale and HPV testing knowledge scale in a sample of Canadian women. Prev. Med. Rep..

[16] Ergöz Aksoy S.Z., Bilgiç D. (2024). Psychometric properties of the Turkish version of the Cervical Cancer Knowledge Scale. J. Obstet. Gynaecol. Res..

[17] McRee A.L., Brewer N.T., Reiter P.L., Gottlieb S.L., Smith J.S. (2010). The Carolina HPV immunization attitudes and beliefs scale (CHIAS): Scale development and associations with intentions to vaccinate. Sex. Transm. Dis..

[18] Sunar S., Kahyaoğlu Süt H. (2019). Turkish validity and reliability study of the Carolina HPV Immunization Attitudes and Beliefs Scale. Jinekoloji-Obstet. Neonatoloji Tıp Derg..

[19] Tabachnick B.G., Fidell L.S. (2019). Using Multivariate Statistics.

[20] Thahirabanuibrahim, Logaraj M. (2023). The effect of the health belief model education for cervical cancer prevention, screening promotion among rural women in Chengalpattu district, Tamil Nadu (HBMECC). J. Educ. Health Promot..

[21] Abdelmonsef Ahmed H.A., Ibrahim Yassin S.Y., Mohamed Ahmed M.S., Yousof Ali H.Z. (2023). Application of Health Belief Model About Cervical Cancer Screening Among Female Officer Employees in Kafr-El Sheikh University, Egypt. JPMA. J. Pak. Med. Assoc..

[22] Fukuda T., Ueda M., Aida R., Ota K., Yoshida H., Shintani A., Okada M., Takaki Y., Amano K., Sumi T. (2024). Educational Interventions to Improve Knowledge and Attitudes Toward Human Papillomavirus (HPV) Vaccination and Cervical Cancer Screening Among Japanese University Students. Cureus.

[23] Chawla P.C., Chawla A.K., Chaudhary S., Chaudhary A. (2021). Knowledge, attitude and practice on human papillomavirus vaccination and cervical cancer screening among healthcare providers. J. Fam. Med. Prim. Care.

[24] Saei Ghare Naz M., Kariman N., Ebadi A., Ozgoli G., Ghasemi V., Rashidi Fakari F. (2018). Educational Interventions for Cervical Cancer Screening Behavior of Women: A Systematic Review. Asian Pac. J. Cancer Prev..

[25] Al-Naggar R.A., Low W.Y., Isa Z.M. (2010). Knowledge and barriers towards cervical cancer screening among young women in Malaysia. Asian Pac. J. Cancer Prev..

[26] Jin S.W., Lee Y., Brandt H.M. (2023). Human Papillomavirus (HPV) Vaccination Knowledge, Beliefs, and Hesitancy Associated with Stages of Parental Readiness for Adolescent HPV Vaccination: Implications for HPV Vaccination Promotion. Trop. Med. Infect. Dis..

[27] Escoffery C., Petagna C., Agnone C., Perez S., Saber L.B., Ryan G., Dhir M., Sekar S., Yeager K.A., Biddell C.B. (2023). A systematic review of interventions to promote HPV vaccination globally. BMC Public Health.

[28] Stephens E.S., Dema E., McGee-Avila J.K., Shiels M.S., Kreimer A.R., Shing J.Z. (2023). Human Papillomavirus Awareness by Educational Level and by Race and Ethnicity. JAMA Netw. Open.

[29] Krawczyk A., Perez S., King L., Vivion M., Dubé E., Rosberger Z. (2015). Parents’ decision-making about the human papillomavirus vaccine for their daughters: II. Qualitative results. Hum. Vaccines Immunother..

[30] Olaoye O., Macdonald S. (2024). A systematic review of interventions to promote human papillomavirus (HPV) vaccination in Africa. Public Health.

[31] Waller J., Marlow L.A., Wardle J. (2007). The association between knowledge of HPV and feelings of stigma, shame and anxiety. Sex. Transm. Infect..

